# Supplementary material for: Validating the validation: reanalyzing a large-scale comparison of deep learning and machine learning models for bioactivity prediction
Source: J Comput Aided Mol Des. 2020 Jan 20;34(7):717–30. doi: 10.1007/s10822-019-00274-0 (PMC7292817; doi:10.1007/s10822-019-00274-0)
Supplement: Supplementary file 1 — Electronic supplementary material 1 (PDF 1233 kb) [file 10822_2019_274_MOESM1_ESM.pdf]

Supplementary Information

Matthew C. Robinson[1], Robert C. Glen[1], Alpha A. Lee[1,]

1. AUC-ROC vs. AUC-PRC Simulations

Below we replicate the computational experiments of Saito and Rehmsmeir from their article "The Precision-Recall Plot is More Informative than the ROC Plot When Evaluating Binary Classifiers on Imbalanced Datasets" [1]. All code for these simulations is provided in the linked GitHub repository.

We begin with their example of "poor early retrieval," which involves the theoretical classifier with scores for positive and negative samples shown in figure 1. As can be seen, positive samples dominate in the region of higher scores; however, a significant number of negative samples are also present in this region. Therefore, classification in the region of high scores (those that would be tested for activity first) will be imperfect.

Figure 2 shows the ROC, PRC, and enrichment factor plots calculated after randomly sampling from the score distributions in figure 1. As in our prior simulations, 100 positive samples and 10,000 negative samples are drawn to simulate a 1% "hit rate" – resembling the class imbalance seen in virtual screens. The ROC plot seems to show good performance while the PRC plot shows poor performance due to the high number of false positives.

Email address: aal44@cam.ac.uk (Alpha A. Lee)

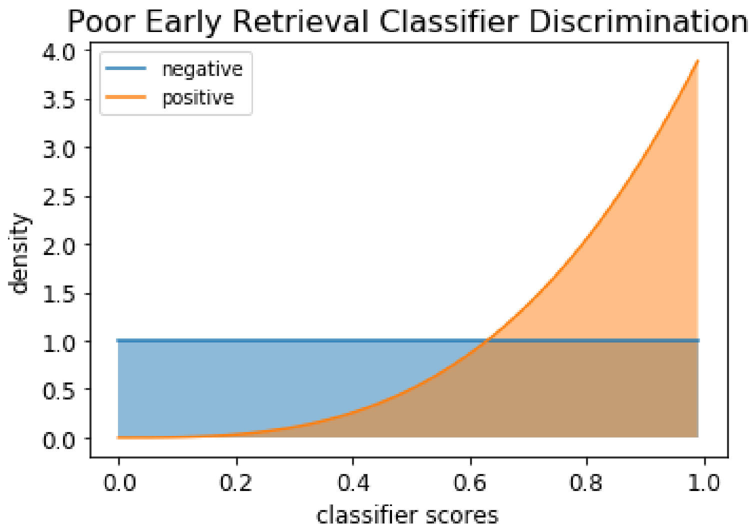

Figure 1: Distributions of classification scores for a theoretical classifier of positive and negative examples. The negative scores follow a $B(a = 1, b = 1)$ distribution, while the positive scores follow a $B(a = 4, b = 1)$ distribution. Adapted from the simulations in [1]

.

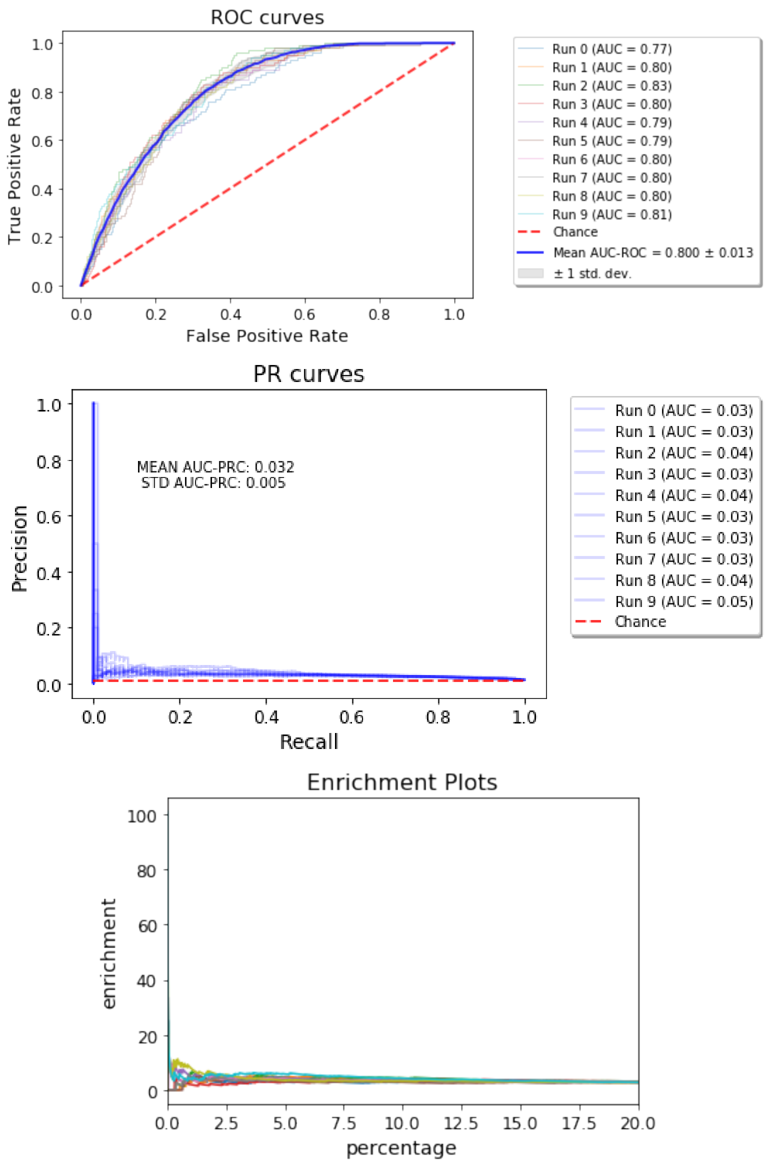

Figure 2: ROC, PRC, and enrichment factor curves for predictions from the theoretical classifier shown in 1. The curves result from 10 runs of a simulation with large class imbalance ($\sim 1\%$ actives)

In contrast, the example of "good early retrieval" in the original paper is given in figures 3 and 4. As can be seen, the AUC-ROC score is almost the same as in the case of "poor early retrieval." The precision-recall and enrichment scores, meanwhile, are much higher than previously.

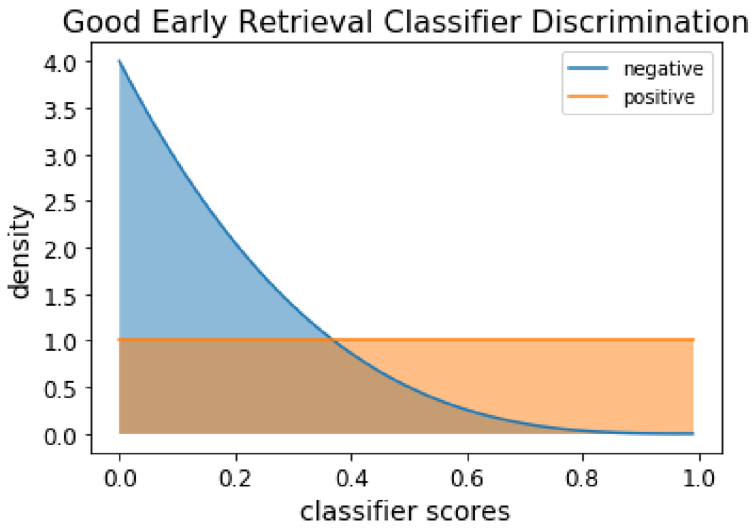

Figure 3: Distributions of classification scores for a theoretical classifier of positive and negative examples. The negative scores follow a $B(a = 1, b = 4)$ distribution, while the positive scores follow a $B(a = 1, b = 1)$ distribution. Adapted from the simulations in [1]

.

Finally, we refer the reader to the provided code notebooks or the original Saito and Rehmsmeir paper for an example of the lack of change in AUC-ROC as compared to AUC-PRC in the event of changing data imbalances.

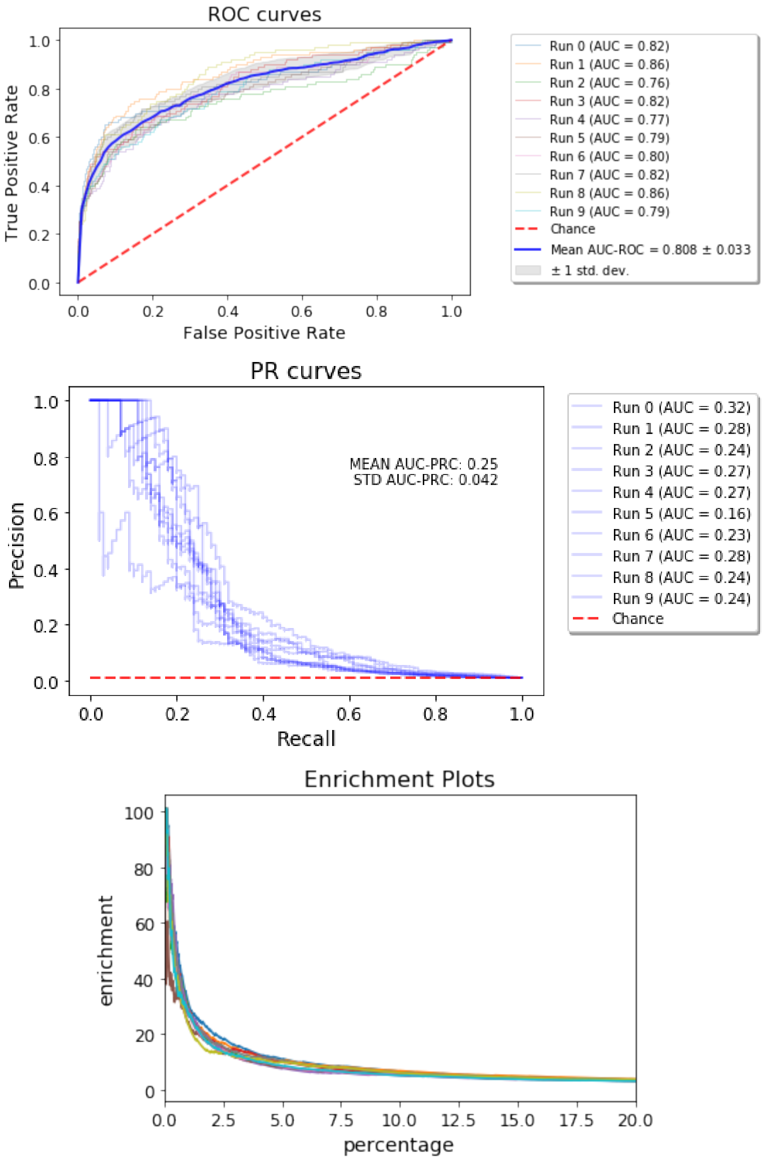

Figure 4: ROC, PRC, and enrichment factor curves for predictions from the theoretical classifier shown in 3. The curves result from 10 runs of a simulation with large class imbalance ($\sim 1\%$ actives)

2. CV Simulations

Adapting the simulations from [2], we performed the simulations described in the text for $N_{train} = 30, 100, 300, 1000$. The results are shown in figure 5. As is expected, the errors in estimating generalization performance from a cross-validation procedure decrease as the sample size increases.

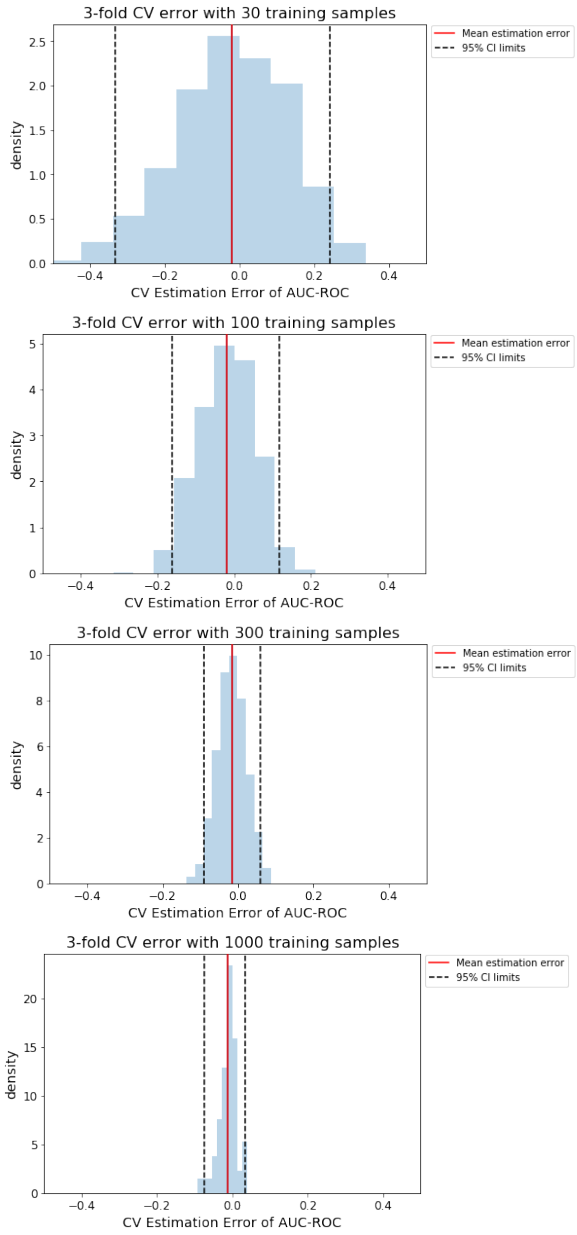

Figure 5: The error in estimating generalization performance from 3-fold cross validation, using the procedure described in the main text.

3. Correlation of Model Mean AUC Performance

In figure 7 we provide the correlation between mean AUC-ROC performances for all models. This differs from the plot in the main text, which only includes the performance on large assays. As is expected, the results are much more variable when including the many assays with small sample sizes. Additionally, we provide the correlation matrix corresponding to figure 7.

	FNN	SVM	RF	NB	KNN	WEAVE	GC	LSTM
FNN	1	0.844289	0.759925	0.704599	0.80311	0.837093	0.855634	0.888644
SVM	0.844289	1	0.863207	0.759618	0.849574	0.817922	0.856315	0.872883
RF	0.759925	0.863207	1	0.697126	0.81876	0.775566	0.779063	0.79362
NB	0.704599	0.759618	0.697126	1	0.70505	0.683644	0.702378	0.700951
KNN	0.80311	0.849574	0.81876	0.70505	1	0.770792	0.800071	0.822428
WEAVE	0.837093	0.817922	0.775566	0.683644	0.770792	1	0.855179	0.874194
GC	0.855634	0.856315	0.779063	0.702378	0.800071	0.855179	1	0.87396
LSTM	0.888644	0.872883	0.79362	0.700951	0.822428	0.874194	0.87396	1

Figure 6: The correlation (Pearson) matrix of all model mean AUC-ROC performances for all assays. This table summarizes the information shown graphically in figure 7

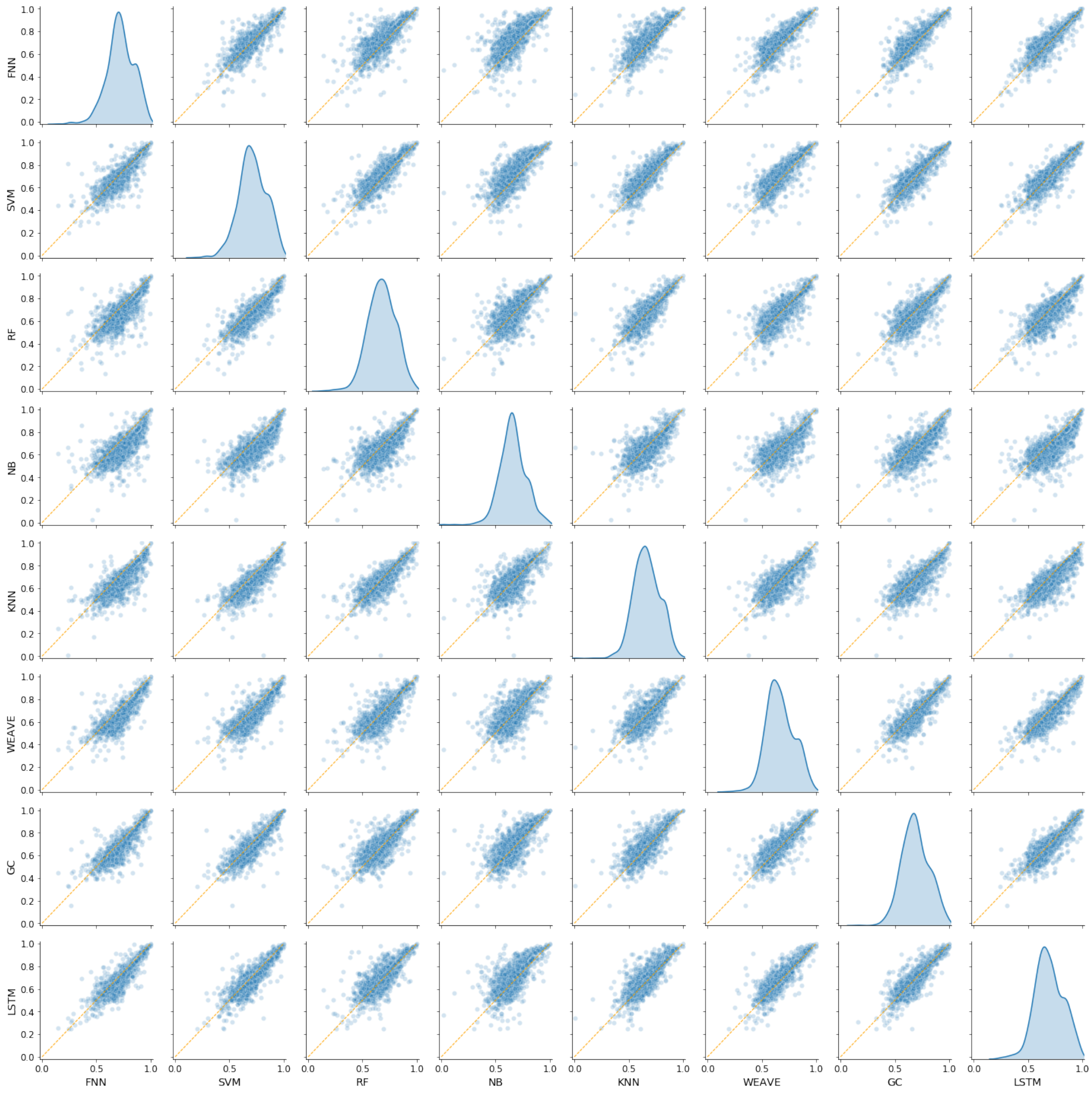

Figure 7: The correlation of all model mean AUC-ROC performances for all assays. The density plots on the diagonal represent the distribution of mean AUC-ROC prediction scores for a given classifier.

4. Additional Plots of Comparative FNN performance

Below are three plots showing the AUC-ROC performance of feed-forward neural networks (FNN) against the performance of three popular "shallow learning" techniques on all test folds. As one can see, performance is highly variable in small sample size assays, while FNN appears to generally outperform the other models on large assays.

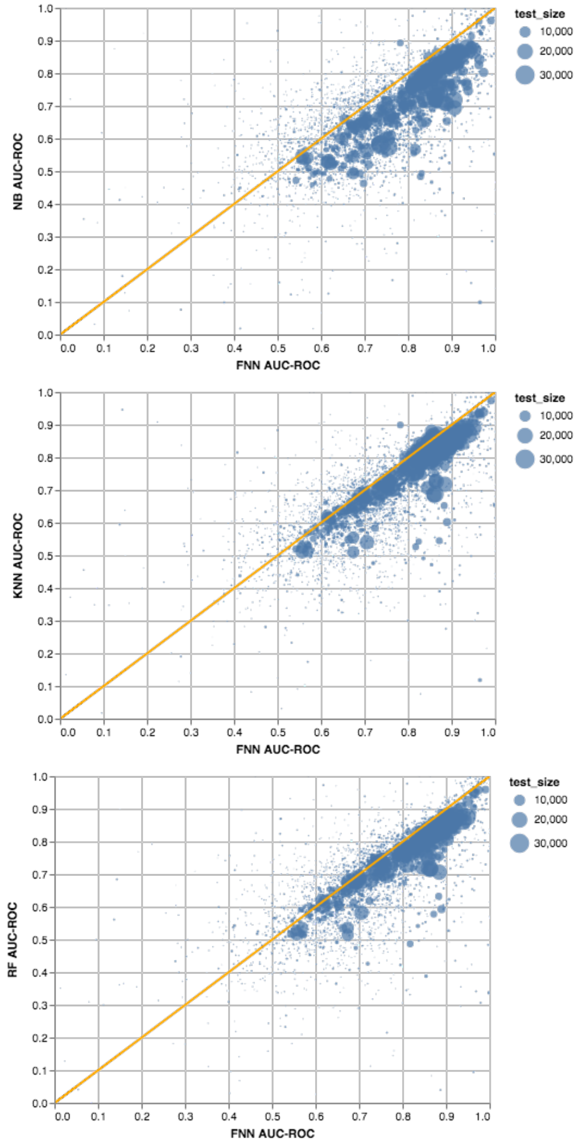

Figure 8: The comparison of AUC-ROC performance on all test folds of FNN against Naive Bayes (NB), k-nearest neighbors (KNN), and random forests (RF)

5. Conclusion

All of the code used for constructing figures and performing data analysis can be found in the following GitHub repository: `https://github.com/mc-robinson/mayr_reanalysis_supp_info`.

[1] T. Saito, M. Rehmsmeier, The precision-recall plot is more informative than the roc plot when evaluating binary classifiers on imbalanced datasets, PloS one 10 (3) (2015) e0118432.

[2] G. Varoquaux, Cross-validation failure: small sample sizes lead to large error bars, Neuroimage 180 (2018) 68–77.
